# Associations Between *APOC3* and *ANGPTL8* Gene Polymorphisms With MASLD Risk and the Mediation Effect of Triglyceride on MASLD in the Chinese Population

**DOI:** 10.1111/jcmm.70542

**Published:** 2025-04-07

**Authors:** Jia Pan, Xue Wang, Youjin Zhang, Ting Wang, Saiqun Lv, Xiaoli Zhang, Yuanyuan Zhou, Tao Peng, Yongyan Song

**Affiliations:** ^1^ Department of Health Management Center Clinical Medical College & Affiliated Hospital of Chengdu University Chengdu Sichuan People's Republic of China; ^2^ Department of Central Laboratory Clinical Medical College & Affiliated Hospital of Chengdu University Chengdu Sichuan People's Republic of China; ^3^ Department of Radiology Clinical Medical College & Affiliated Hospital of Chengdu University Chengdu Sichuan People's Republic of China; ^4^ Institute of Basic Medicine and Forensic Medicine North Sichuan Medical College Nanchong People's Republic of China

**Keywords:** *ANGPTL*8, *APOC3*, mediation analysis, metabolic‐associated liver disease, polymorphisms, triglycerides

## Abstract

Apolipoprotein C3 (*APOC3*) and angiopoietin‐like protein 8 (*ANGPTL*8) genes are related to lipid metabolism. The relationships between single nucleotide polymorphisms (SNPs) in the *APOC3* and *ANGPTL*8 genes with metabolic dysfunction‐associated steatotic liver disease (MASLD) remain controversial. This study aimed to investigate the associations between specific SNPs in the *APOC3* and *ANGPTL*8 genes and MASLD risk, with a particular focus on the mediating role of triglycerides (TG). A total of 440 participants were enrolled and categorised into MASLD and control groups. Genotyping of *APOC3* SNPs (rs5128, rs2854116 and rs2854117) and *ANGPTL*8 SNP (rs2278426) was conducted using polymerase chain reaction–restriction fragment length polymorphism or Sanger sequencing methods. Multivariate logistic regression was employed to estimate the associations between these SNPs and MASLD risk, and mediation analysis was performed to assess the potential mediating effect of TG. We found that *APOC3* SNPs were associated with MASLD risk, with increased odds ratios (ORs) indicating a higher risk of MASLD: rs5128 CG + GG genotype (OR = 1.8, 95% CI = 1.1–2.8), rs2854116 TC + CC genotype (OR = 1.9, 95% CI = 1.1–3.1) and rs2854117 CT + TT genotype (OR = 1.9, 95% CI = 1.2–3.2). No association was found between *ANGPTL*8 rs2278426 and MASLD (*p* > 0.05). Mediation analysis revealed that TG significantly mediated these relationships, accounting for 80.25% of the effect for rs5128, 64.61% for rs2854116 and 62.59% for rs2854117. In summary, polymorphisms in *APOC3* (rs5128, rs2854116 and rs2854117) were associated with MASLD risk, with TG serving as a potential mediating factor. In contrast, *ANGPTL*8 rs2278426 polymorphism did not show any association with MASLD.

## 1 Introduction

Metabolic dysfunction‐associated steatotic liver disease (MASLD) has emerged as the leading cause of chronic liver disease globally, affecting approximately 25%–38% of the population [1, 2]. The reasons for the variations in prevalence are unknown, but it is speculated that genetic and lifestyle factors may play a major role. Some studies suggested that genetic factors account for about half of MASLD patients [3].

Increasing evidence supported the importance of single nucleotide polymorphisms (SNPs) in the risk of MASLD, particularly those found in genes related to lipid metabolism, such as apolipoprotein C3 (*APOC3*) and angiopoietin‐like protein 8 (*ANGPTL*8) [4, 5]. The *APOC3* gene is involved in lipid metabolism, particularly in triglyceride (TG) metabolism [6]. Previous studies showed that *APOC3* SNPs, such as rs5128, rs2854116 and rs2854117, are closely related to dyslipidaemia and hepatic fat accumulation [7, 8]. However, the results were controversial in different studies [7, 9]. Furthermore, there are limited studies of gene polymorphisms affecting the occurrence of MASLD through mediating factors. More studies must be carried out.

*ANGPTL*8 is widely expressed in hepatocytes and plays a crucial role in lipid metabolism and insulin regulation [10]. Both animal and human studies have demonstrated higher levels of *ANGPTL*8 in MASLD patients [11]. Meanwhile, there are very few articles examining the associations of the *ANGPTL*8 SNPs with MASLD. The *ANGPTL*8 rs2278426 is a SNP locus that has been identified in recent years [12]. The association of this SNP with MASLD has only been investigated in a study involving an Iranian population, with a lack of data from China [13].

Therefore, this study aims to explore the associations between *APOC3* SNPs (rs5128, rs2854116 and rs2854117) and *ANGPTL*8 SNP (rs2278426) with MASLD susceptibility in the Chinese population.

## 2 Materials and Methods

### 2.1 Study Subjects

A total of 440 participants were enrolled from the Department of Health Management Center of Clinical Medical College & Affiliated Hospital of Chengdu University between April and October 2022. Participants eligible for this study had to meet the following criteria: (1) aged 18 years or older; (2) individuals diagnosed with MASLD according to the latest criteria [14] were included in the MASLD group. Healthy control participants were required to have normal liver function tests and no evidence of liver disease; (3) volunteer to participate in the study and complete relevant medical examinations. Exclusion criteria: (1) a history of systemic diseases (e.g., stroke, thyroid disorder, liver or renal failure and malignant) and (2) incomplete relevant medical examinations. This study was approved by the Ethics Committee of Clinical Medical College & Affiliated Hospital of Chengdu University (PJ2024‐097‐02).

### 2.2 Epidemiological Survey and Biochemical Measurements

All participants answered standardised questionnaires about age, sex, medical history and history of smoking and drinking. Blood pressure (BP) was measured with electronic sphygmomanometers. Body mass index (BMI) was calculated as weight (in kilogrammes) divided by height (in metres) squared. All participants were routinely examined with upper abdominal ultrasonography. Blood samples were tested by laboratory physicians, including measurements of alanine aminotransferase (ALT), aspartate aminotransferase (AST), γ‐glutamyl transpeptidase (GGT), alkaline phosphatase (ALP), total bilirubin (TB), globulin (GLB), albumin (ALB), total protein (TP), cholesterol (CHO), TG, low‐density lipoprotein cholesterol (LDL‐C), high‐density lipoprotein cholesterol (HDL‐C), creatinine (Cr), uric acid (UA), fasting blood glucose (FBG), haemoglobin A1c (HbA1c), platelet (PLT) and C‐reactive protein (CRP).

### 2.3 Genomic DNA Extraction and Genotyping

Genomic DNA was extracted from peripheral blood using the Tiangen DNA Kit (Tiangen Biotech, Beijing, China) according to the manufacturer's instructions. Genotyping of the *APOC3* gene rs5128 and *ANGPTL*8 rs2278426 was performed using polymerase chain reaction followed by polymerase chain reaction–restriction fragment length polymorphism (PCR‐RFLP). The PCR products were digested by the restriction endonucleases. Next, the DNA fragments were separated on agarose gel. For the *APOC3* rs2854116 and rs2854117, genotyping was performed using Sanger sequencing (Thermo Fisher Scientific 3730xl DNA Analyser). The sequences of primers are listed in Table 1, and the PCR and PCR‐RFLP protocols are listed in Supplement 1.

**TABLE 1:** Primer sequences for *APOC3* and *ANGPTL*8 genotyping.

Primer description	Sequence
*APOC3* rs2854116 primer	Forward: GAAACCCAGAGATGGAGGTG Reverse: TCTCAGCCTTTCACACTGGA
*APOC3* rs2854117 primer	Forward: GAAACCCAGAGATGGAGGTG Reverse: TCTCAGCCTTTCACACTGGA
*APOC3* rs5128 PCR primer	Forward: GGTGACCGATGGCTTCAGTTCCCTGA Reverse: CAGAAGGTGGATAGAGCGCTGGCC
*ANGPTL*8 rs2278426 PCR primer	Forward: CAGGAGTTCTATTGTGCGGC Reverse: CCTGATGCAACTATCGCACC

### 2.4 Statistical Analysis

Continuous data were expressed as mean ± standard deviation. Categorical variables were presented as frequencies and percentages. Comparisons between groups were performed using independent samples t‐test and the chi‐square test. Multivariable logistic regression analysis was employed to evaluate the associations between *APOC3* and *ANGPTL*8 SNPs and the risk of MASLD, adjusting for potential confounders. Odds ratios (ORs) and corresponding 95% confidence intervals (CIs) were obtained to evaluate the effect. Mediation analysis was performed to assess the mediating effect of TG on the relationship between SNPs and MASLD. All analytical processes were conducted utilising R package version 4.2.0 and EmpowerStats version 4.2. A statistically significant result was determined as a two‐sided *p* value < 0.05.

## 3 Results

### 3.1 Characteristics of the Study Participants

A total of 440 participants were enrolled in the study. The baseline characteristics of the participants are shown in Table 2. The levels of BMI, BP, ALT, AST, GGT, ALP, ALB, TG, HDL‐C, UA, FBG and HbA1c were higher in the MASLD group as compared to the control group (*p* < 0.05). There were no significant differences in age, history of hyperlipidaemia, smoking habits or levels of TB, GLB, TP, Cr, CHO, LDL‐C, PLT and CRP between the two groups (*p* > 0.05).

**TABLE 2:** Clinical characteristics of the study population.

Variables	Control group (*N* = 221)	MASLD group (*N* = 219)	*p*
Demographic variables			
Age, years	53.94 ± 10.44	53.57 ± 9.87	0.699
Male/female	139/82	183/36	< 0.001
Medical history			
Hypertension, *n* (%)	30 (16.13)	70 (34.65)	< 0.001
Diabetes, *n* (%)	11 (5.91)	34 (16.83)	< 0.001
Hyperlipidaemia, *n* (%)	7 (3.76)	14 (6.93)	0.168
Lifestyle behaviours			
Smoking, *n* (%)	69 (37.10)	92 (45.77)	0.084
Drinking, *n* (%)	81 (43.55)	120 (59.70)	0.001
Clinical variables			
BMI, kg/m^2^	23.04 ± 2.76	26.29 ± 3.15	< 0.001
SBP, mmHg	119.04 ± 15.96	130.32 ± 17.72	< 0.001
DBP, mmHg	74.29 ± 10.82	82.36 ± 11.01	< 0.001
ALT, U/L	23.43 ± 12.56	38.04 ± 23.99	< 0.001
AST, U/L	24.56 ± 7.17	30.78 ± 24.21	< 0.001
GGT, U/L	24.99 ± 22.41	51.87 ± 51.66	< 0.001
ALP, U/L	67.23 ± 16.52	75.87 ± 27.10	0.007
TB, umol/L	15.94 ± 5.33	18.44 ± 18.48	0.057
GLB, g/L	26.90 ± 3.20	26.76 ± 3.87	0.674
ALB, g/L	44.40 ± 3.03	45.10 ± 3.59	0.031
TP, g/L	71.31 ± 4.41	71.86 ± 5.16	0.236
CHO, mmol/L	5.04 ± 1.01	4.91 ± 0.94	0.163
TG, mmol/L	1.67 ± 1.44	2.57 ± 1.90	< 0.001
HDL‐C, mmol/L	1.74 ± 0.43	1.44 ± 0.36	< 0.001
LDL‐C, mmol/L	2.71 ± 0.71	2.76 ± 0.68	0.426
Cr, mmol/L	69.86 ± 29.77	70.43 ± 15.67	0.805
UA, μmol/L	339.49 ± 85.79	405.50 ± 107.55	< 0.001
FBG, mmol/L	5.32 ± 1.35	6.08 ± 1.90	< 0.001
HbA1c, %	5.90 ± 0.48	6.37 ± 0.99	< 0.001
PLT, 10^9^/L	201.34 ± 57.65	196.54 ± 60.04	0.393
CRP, mg/L	3.30 ± 12.10	6.31 ± 16.25	0.074

Abbreviations: ALB, albumin; ALP, alkaline phosphatase; ALT, alanine aminotransferase; AST, aspartate aminotransferase; BMI, body mass index; CHO, cholesterol; Cr, creatinine; CRP, C‐reactive protein; DBP, diastolic blood pressure; FBG, fasting blood glucose; GGT, γ‐glutamyl transpeptidase; GLB, globulin; HbA1c, haemoglobin A1c; HDL‐C, high‐density lipoprotein cholesterol; LDL‐C, low‐density lipoprotein cholesterol; MASLD, Metabolic dysfunction‐associated steatotic liver disease; PLT, platelet; SBP, systolic blood pressure; TB, total bilirubin; TG, triglyceride; TP, total protein; UA, uric acid.

### 3.2 Genetic Characteristics of the Study Population

**FIGURE 1:**
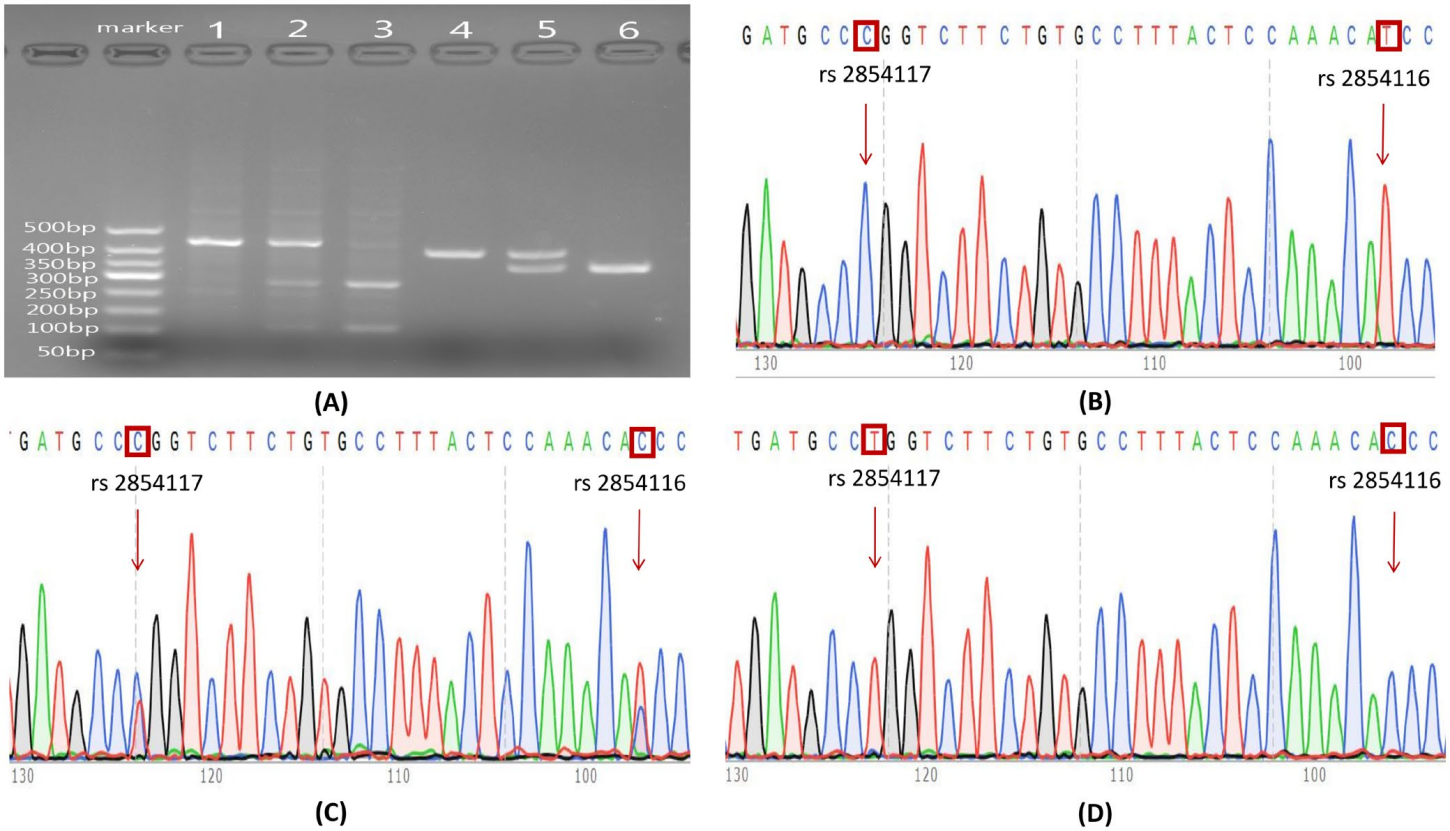
Genotyping results of *APOC*3 SNPs (rs5128, rs2854116 and rs2854117) and *ANGPTL*8 SNP (rs2278426). (A) Genotyping of *APOC*3 rs5128 and *ANGPTL*8 rs2278426 was performed using PCR‐RFLP. Lane 1 (rs5128): CC genotype; Lane 2 (rs5128): GC genotype; Lane 3 (rs5128): GG genotype; Lane 4 (rs2278426): CC genotype; Lane 5: CT genotype (rs2278426); and Lane 6 (rs2278426): TT genotype. Genotyping of *APOC*3 rs2854116 and rs2854117 was performed using Sanger sequencing. (B) rs2854116: TT genotype; rs2854117: CC genotype; (C) rs2854116: TC genotype; rs2854117: CT genotype; (D) rs2854116: CC genotype; rs2854117: TT genotype.

The genotyping results of *APOC*3 SNPs (rs5128, rs2854116 and rs2854117) and *ANGPTL*8 SNP (rs2278426) are illustrated in Figure 1.

As shown in Table 3, significant differences were observed in the distribution of alleles or genotypes for *APOC3* rs5128, rs2854116 and rs2854117 between the control group and the MASLD group. For *APOC3* rs5128, the CG + GG genotype was more prevalent in the MASLD group (50.68%) compared to the control group (39.82%) (*p* = 0.022). The frequency of the G allele was also higher in the MASLD group as compared to the control group (*p* = 0.023). For *APOC3* rs2854116, the CT + CC genotype was more prevalent in the MASLD group (71.23%) than in the control group (61.09%), with a significant *p* value of 0.025. The frequency of the C allele showed a higher frequency in the MASLD group compared to the control group. The trends for *APOC3* rs2854117 were similar to those of rs2854116. However, for the *ANGPTL*8 rs2278426, no significant differences were observed in the distribution of different genotypes or alleles.

**TABLE 3:** Genetic characteristics of the study population.

SNPs	Control group (*N* = 221)	MASLD group (*N* = 219)	*p*
*APOC3* rs5128			
CC, *n* (%)	133 (60.18)	108 (49.32)	0.049
CG, *n* (%)	83 (37.56)	101 (46.12)	
GG, *n* (%)	5 (2.26)	10 (4.57)	
CG + GG, *n* (%)	88 (39.82)	111 (50.68)	0.022
C allele	0.79	0.72	0.023
G allele	0.21	0.27	
*APOC3* rs2854116			0.062
TT, *n* (%)	86 (38.91)	63 (28.77)	
CT, *n* (%)	115 (52.04)	128 (58.45)	
CC, *n* (%)	20 (9.05)	28 (12.79)	
CT + CC, *n* (%)	135 (61.09)	156 (71.23)	0.025
T allele	0.65	0.58	0.034
C allele	0.35	0.42	
*APOC3* rs2854117			
CC, *n* (%)	90 (40.72)	62 (28.31)	0.016
CT, *n* (%)	112 (50.68)	128 (58.45)	
TT, *n* (%)	19 (8.60)	29 (13.24)	
CT + TT, *n* (%)	131 (59.28)	157 (71.69)	0.006
C allele	0.66	0.58	0.009
T allele	0.34	0.43	
*ANGPTL*8 rs2278426			
CC, *n* (%)	119 (53.85)	115 (52.51)	0.931
TC, *n* (%)	86 (38.91)	89 (40.64)	
TT, *n* (%)	16 (7.24)	15 (6.85)	
TC + TT, *n* (%)	102 (46.15)	104 (47.49)	0.779
C allele	0.73	0.73	0.875
T allele	0.27	0.27	

Abbreviations: ANGPTL8, angiopoietin‐like 8; *APOC*3, apolipoprotein C‐III; MASLD, Metabolic dysfunction‐associated steatotic liver disease; SNP, single nucleotide polymorphism.

### 3.3 Metabolic Variables of the Participants According to the *APOC3* and *ANGPTL*8 Genotypes

Given that MASLD is closely related to metabolic function, we analysed the metabolic variables among different SNP genotypes. In the *APOC3* rs5128 genotypes, participants with CG or GG genotypes exhibited significantly higher TG levels compared to those with the CC genotype in both the control and MASLD groups (*p* < 0.05). In the control group, the levels of FBG and HbA1c were higher in the GG genotype compared to the CC and CG genotypes (Table 4). The results of the rs2854117 and rs2854116 polymorphisms were similar to the rs5128 genotype, while the *ANGPTL*8 rs2278426 SNP result remained statistically nonsignificant (Tables 5 and 6).

**TABLE 4:** Metabolic variables of the participants for the *APOC*3 rs5128 genotype.

Metabolic variables	Control group				MASLD group			
	CC genotype (*N* = 133)	CG genotype (*N* = 83)	GG genotype (*N* = 5)	*p*	CC genotype (*N* = 108)	CG genotype (*N* = 101)	GG genotype (*N* = 10)	*p*
BMI, kg/m^2^	22.87 ± 2.69	23.33 ± 2.89	22.79 ± 2.52	0.522	26.00 ± 3.18	26.62 ± 3.15	26.08 ± 2.84	0.386
SBP, mmHg	117.96 ± 15.43	120.96 ± 16.91	114.67 ± 10.97	0.399	128.99 ± 18.67	131.37 ± 16.78	133.56 ± 17.80	0.546
DBP, mmHg	73.33 ± 10.75	75.93 ± 10.84	72.33 ± 11.59	0.255	82.34 ± 11.27	82.36 ± 10.98	82.67 ± 9.14	0.996
CHO, mmol/L	4.98 ± 1.01^a^	5.05 ± 0.94^b^	6.20 ± 1.49^b^	0.029	4.99 ± 0.85	4.86 ± 1.03	4.35 ± 0.87	0.122
TG, mmol/L	1.52 ± 1.15^a^	1.67 ± 1.44^a^	5.39 ± 2.80^b^	< 0.001	2.17 ± 1.40^a^	3.00 ± 2.28^bc^	2.67 ± 1.48^ac^	0.007
HDL‐C, mmol/L	1.77 ± 0.44	1.73 ± 0.42	1.30 ± 0.20	0.059	1.50 ± 0.34	1.39 ± 0.38	1.45 ± 0.36	0.086
LDL‐C, mmol/L	2.67 ± 0.70	2.74 ± 0.70	3.37 ± 0.77	0.083	2.83 ± 0.59	2.73 ± 0.75	2.35 ± 0.74	0.097
UA, μmol/L	340.42 ± 85.16	336.40 ± 87.90	364.00 ± 80.09	0.770	412.07 ± 106.14	400.97 ± 103.39	376.77 ± 165.31	0.547
FBG, mmol/L	5.47 ± 1.59^a^	5.00 ± 0.56^b^	6.74 ± 2.21^c^	0.003	6.24 ± 2.09	5.97 ± 1.73	5.31 ± 1.03	0.270
HbA1c, %	5.88 ± 0.39^a^	5.85 ± 0.30^a^	8.05 ± 1.63^b^	< 0.001	6.52 ± 1.17	6.21 ± 0.75	6.14 ± 0.26	0.223

*Note:* ^abc^The same letters within a row indicate no significant difference (*p* > 0.05); different letters indicate significant differences (*p* < 0.05).

Abbreviations: BMI, body mass index; CHO, cholesterol; DBP, diastolic blood pressure; FBG, fasting blood glucose; HbA1C, haemoglobin A1c; HDL‐C, high‐density lipoprotein cholesterol; LDL‐C, low‐density lipoprotein cholesterol; MASLD, Metabolic dysfunction‐associated steatotic liver disease; SBP, systolic blood pressure; TG, triglyceride; UA, uric acid.

**TABLE 5:** Metabolic variables of the participants for the *APOC*3 rs2854116 genotype.

	Control group				MASLD group			
Metabolic variables	TT genotype (*N* = 86)	TC genotype (*N* = 115)	CC genotype (*N* = 20)	*p*	TT genotype (*N* = 63)	TC genotype (*N* = 128)	CC genotype (*N* = 28)	*p*
BMI, kg/m^2^	23.11 ± 2.67	23.08 ± 2.90	22.40 ± 2.36	0.646	26.47 ± 3.10	26.11 ± 3.16	26.84 ± 3.28	0.506
SBP, mmHg	120.95 ± 16.13	118.19 ± 16.33	114.60 ± 11.19	0.274	132.09 ± 21.70	129.15 ± 15.54	132.04 ± 18.08	0.506
DBP, mmHg	75.74 ± 11.22	73.32 ± 10.70	73.20 ± 9.10	0.303	83.21 ± 11.63	81.84 ± 10.40	83.00 ± 12.68	0.703
CHO, mmol/L	5.09 ± 0.96^a^	4.87 ± 0.97^a^	5.75 ± 1.17^b^	0.001	5.07 ± 0.89	4.87 ± 0.94	4.71 ± 1.08	0.201
TG, mmol/L	1.51 ± 1.08^a^	1.52 ± 1.13^a^	3.12 ± 2.84^b^	< 0.001	2.08 ± 1.37^a^	2.63 ± 1.81^a^	3.44 ± 2.88^b^	0.007
HDL‐C, mmol/L	1.82 ± 0.44	1.71 ± 0.42	1.62 ± 0.43	0.108	1.49 ± 0.34	1.44 ± 0.36	1.33 ± 0.38	0.172
LDL‐C, mmol/L	2.72 ± 0.68^a^	2.62 ± 0.69^a^	3.19 ± 0.76^b^	0.003	2.90 ± 0.61	2.74 ± 0.69	2.56 ± 0.71	0.081
UA, μmol/L	341.27 ± 89.25	339.29 ± 81.11	333.15 ± 99.77	0.930	409.64 ± 113.52	397.61 ± 102.08	433.88 ± 117.49	0.276
FBG, mmol/L	5.33 ± 0.99	5.29 ± 1.57	5.44 ± 1.36	0.904	6.42 ± 2.28	5.90 ± 1.70	6.11 ± 1.80	0.217
HbA1c, %	5.90 ± 0.33^a^	5.84 ± 0.38^a^	6.26 ± 1.06^b^	0.026	6.65 ± 1.31	6.20 ± 0.78	6.47 ± 0.86	0.075

*Note:* ^abc^The same letters within a row indicate no significant difference (*p* > 0.05); different letters indicate significant differences (*p* < 0.05).

Abbreviations: BMI, body mass index; CHO, cholesterol; DBP, diastolic blood pressure; FBG, fasting blood glucose; HbA1C, haemoglobin A1c; HDL‐C, high‐density lipoprotein cholesterol; LDL‐C, low‐density lipoprotein cholesterol; MASLD, Metabolic dysfunction‐associated steatotic liver disease; SBP, systolic blood pressure; TG, triglyceride; UA, uric acid.

**TABLE 6:** Metabolic variables of the participants for the *APOC*3 rs2854117 genotype.

Metabolic variables	Control group				MASLD group			
	CC genotype (*N* = 90)	CT genotype (*N* = 112)	TT genotype (*N* = 19)	*p*	CC genotype (*N* = 62)	CT genotype (*N* = 128)	TT genotype (*N* = 29)	*p*
BMI, kg/m^2^	23.18 ± 2.67	22.96 ± 2.91	22.82 ± 2.36	0.830	26.11 ± 2.84	26.21 ± 3.32	27.05 ± 2.99	0.401
SBP, mmHg	120.96 ± 16.37	117.90 ± 16.13	115.50 ± 11.04	0.300	131.30 ± 21.09	129.25 ± 16.08	133.14 ± 17.68	0.514
DBP, mmHg	75.51 ± 11.13	73.19 ± 10.89	74.79 ± 7.72	0.348	83.00 ± 11.22	82.01 ± 10.68	82.68 ± 12.34	0.844
CHO, mmol/L	5.10 ± 0.94^a^	4.85 ± 0.97^a^	5.79 ± 1.18^b^	< 0.001	4.99 ± 0.89	4.90 ± 0.96	4.75 ± 1.01	0.515
TG, mmol/L	1.52 ± 1.06^a^	1.54 ± 1.17^a^	3.07 ± 2.91^b^	< 0.001	1.91 ± 1.01^a^	2.73 ± 1.89^b^	3.32 ± 2.82^b^	0.002
HDL‐C, mmol/L	1.80 ± 0.45	1.72 ± 0.42	1.60 ± 0.40	0.148	1.51 ± 0.34	1.43 ± 0.37	1.37 ± 0.35	0.191
LDL‐C, mmol/L	2.74 ± 0.67^a^	2.59 ± 0.68^a^	3.24 ± 0.77^b^	< 0.001	2.85 ± 0.64	2.76 ± 0.70	2.60 ± 0.66	0.273
UA, μmol/L	349.50 ± 92.54	331.82 ± 76.60	336.74 ± 101.88	0.355	399.45 ± 98.74	403.68 ± 109.82	426.82 ± 116.73	0.517
FBG, mmol/L	5.30 ± 0.96	5.30 ± 1.60	5.52 ± 1.37	0.800	6.41 ± 2.31	5.92 ± 1.70	6.07 ± 1.75	0.258
HbA1c, %	5.88 ± 0.33^a^	5.86 ± 0.38^a^	6.28 ± 1.12^b^	0.032	6.68 ± 1.30	6.20 ± 0.80	6.44 ± 0.84	0.065

*Note:* ^abc^The same letters within a row indicate no significant difference (*p* > 0.05); different letters indicate significant differences (*p* <  0.05).

Abbreviations: BMI, body mass index; CHO, cholesterol; DBP, diastolic blood pressure; FBG, fasting blood glucose; HbA1C, haemoglobin A1c; HDL‐C, high‐density lipoprotein cholesterol; LDL‐C, low‐density lipoprotein cholesterol; MASLD, Metabolic dysfunction‐associated steatotic liver disease; SBP, systolic blood pressure; TG, triglyceride; UA, uric acid.

### 3.4 Associations Between the *APOC3* and *ANGPTL*8 SNPs and MASLD Risk

**FIGURE 2:**
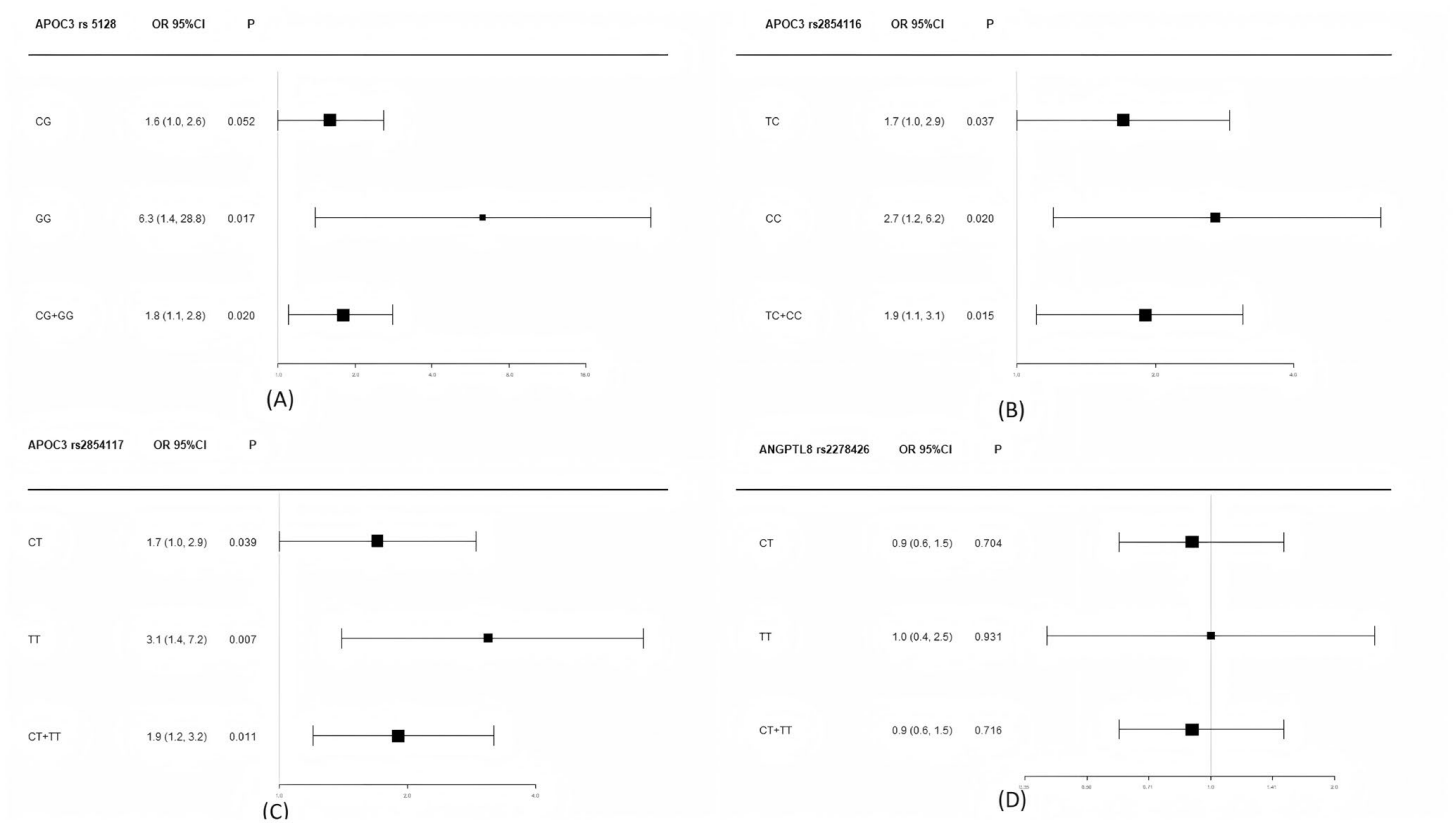
Forest plot analysis of *APOC*3 and *ANGPTL*8 genetic polymorphisms associated with MASLD. (A) The reference group was *APOC*3 rs5128 CC genotype; (B) the reference group was *APOC*3 rs2854116 TT genotype; (C) the reference group was *APOC*3 rs2854117 CC genotype; (D) the reference group was *ANGPTL*8 rs2278426 CC genotype. All the models were adjusted for sex, age, drink, smoking, ALT, AST, CHO and LDL‐C.

As shown in Figure 2, logistic regression analysis was conducted to evaluate the associations of *APOC3* and *ANGPTL*8 SNPs with MASLD after adjusting for sex, age, alcohol drinking, smoking, ALT, AST, CHO and LDL‐C. Compared to the rs5128 CC genotype of *APOC3*, the CG genotype was associated with an OR of 1.6 (95% CI: 1.0–2.6), approaching statistical significance (*p* = 0.052). The GG genotype and the combined genotype of CG + GG exhibited significantly increased ORs (*p* < 0.05). For the *APOC3* rs2854116 SNP, the TC genotype showed an OR of 1.7 (95% CI: 1.0–2.9, *p* = 0.037), the CC genotype had an OR of 2.7 (95% CI: 1.2–6.2, *p* = 0.020) and the combined TC + CC genotype presented an OR of 1.9 (95% CI: 1.1–3.1, *p* = 0.015) compared to the wild genotype. A similar trend was observed for rs2854117, with the OR for the CT genotype at 1.7 (95% CI: 1.0–2.9, *p* = 0.039), the TT genotype at 3.1 (95% CI: 1.4–7.2, *p* = 0.007) and the CT + TT genotype at 1.9 (95% CI: 1.2–3.2, *p* = 0.011). However, no statistical significance was found for the *ANGPTL*8 rs2278426 CT, TT and CT + TT genotypes compared to the CC genotype (*p* > 0.05).

### 3.5 Mediated Effect of TG on the Associations of *APOC*3 SNPs and MASLD

**FIGURE 3:**
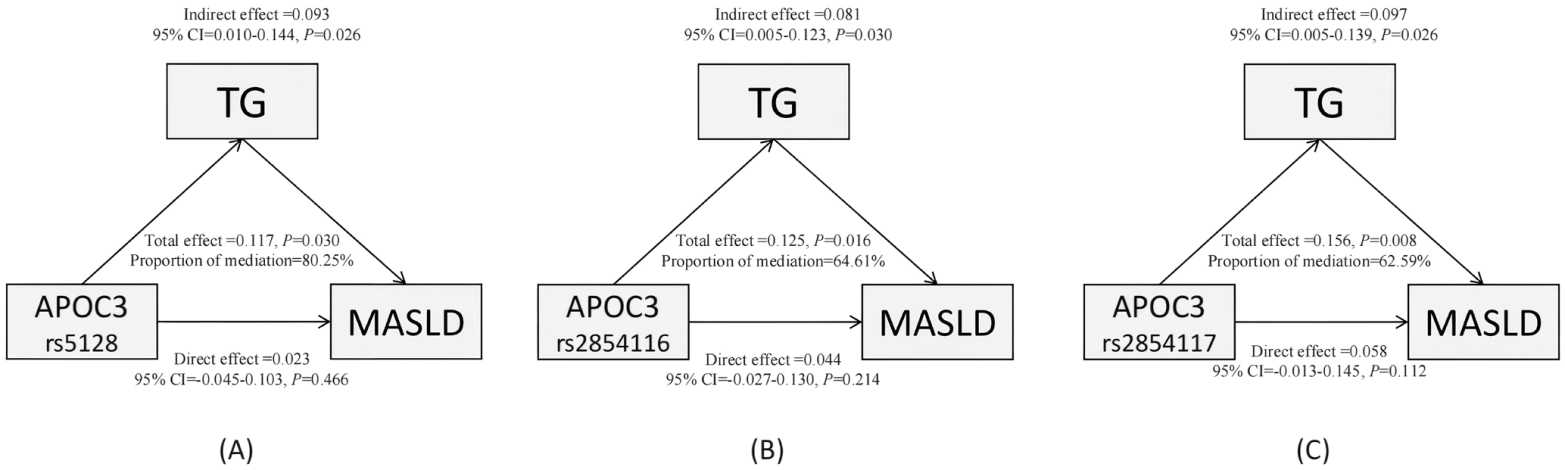
Mediated effect of TG on the associations of APOC3 SNPs and MASLD. Abbreviations: *APOC*3, Apolipoprotein C‐III; TG, triglyceride.

As reported above, the TG levels in the MASLD group were higher than those in the control group. Potential mediation effects of TG were evaluated in the mediation analyses (Figure 3). The results indicated that TG served as a significant mediator of the positive relationship between *APOC3* SNPs and MASLD, with the proportion of mediated effects of 80.25% for rs5128, 64.61% for rs2854116 and 62.59% for rs2854117.

## 4 Discussion

In the present study, we investigated the associations between SNPs in the *APOC3* and *ANGPTL*8 genes and MASLD risk. Our findings suggested that *APOC3* rs5128, rs2854116 and rs2854117 polymorphisms may increase susceptibility to MASLD, with TG serving as a potential mediating factor. This highlights the interplay between genetic factors and lipid metabolism in the development of MASLD. Notably, no association between the *ANGPTL*8 rs2278426 and MASLD risk was found in our study.

Basic research has shown that hepatic overexpression of *APOC3* is prone to the development of MASLD, suggesting that *APOC3* may be a potential biomarker for MASLD [15]. Gene polymorphisms can affect gene expression [16, 17]. The relationship between genetic polymorphisms and gene expression levels is complex, with genetic polymorphisms possessing the potential to modulate gene expression. Both rs2854116 and rs2854117 polymorphisms were located in the insulin response element of the *APOC3* promoter and have been shown to attenuate *APOC3* insulin responsiveness in vitro, subsequently increasing the production of apoC‐III [8]. A clinical study conducted among MASLD patients indicated that *APOC3* variant alleles (rs2854116, rs2854117 or both) increased 30% in the fasting plasma *APOC3* concentration and 60% in TG, as compared with the wild‐type homozygotes [7]. Subsequent studies confirmed this perspective [18]. Our study also demonstrated that the rs2854116 and rs2854117 polymorphisms were associated with MASLD. Contrarily, some studies reported no significant correlation between polymorphisms in the *APOC3* promoter region and MASLD risk [9]. The reasons for these inconsistent conclusions may be attributed to potential heterogeneity in genetic background [8], environmental factors [19] and metabolic conditions [9], thus varying across different populations.

Abnormal accumulation of TG was an important contributor to MASLD [20]. The *APOC3* gene was closely related to TG metabolism [21]. Some mutations that disrupt *APOC3* function were associated with lower levels of plasma TG and *APOC3* [22, 23]. However, there was a debate regarding which mutations result in increased TG levels. Some scholars supported that the rs2854116 and rs2854117 polymorphisms can elevate intrahepatic TG levels [24]. Our study also indicated that individuals with mutations at the rs2854116 and rs2854117 had higher TG levels, although other studies argued that the relationship between the two SNPs was not definitive [9, 25]. Consistent with our results, most studies suggested that rs5128 polymorphisms can increase TG levels [8, 26, 27, 28]. Some research posited that *APOC3* gene polymorphisms may participate in the pathogenesis of MASLD through their effects on triglyceride metabolism [29]. To our knowledge, our study was the first study to explore the mediating effect of TG levels on the association between *APOC3* rs5128, rs2854116 and rs2854117 and MASLD in the Chinese population. There were few studies on *ANGPTL*8 rs2278426 and MASLD, and our findings were not consistent with those of the Iranian population [12]. Our results showed that the mediating effect of TG was higher than that of other polymorphic loci [30], suggesting that these SNPs may provide important clinical significance for the diagnosis and treatment of MASLD.

*APOC3* reduces the hydrolysis and clearance of TG by inhibiting the activity of lipoprotein lipase, increases the number of circulating very‐low‐density lipoprotein particles (VLDL) by promoting the assembly and secretion of VLDL and delays the metabolism of TG‐rich particles by inhibiting the activity of hepatic lipase, eventually leading to impaired TG metabolism [31, 32, 33]. As a key regulator, TG mediates the relationship between *APOC3* polymorphism and the increased risk of MASLD. We hypothesise that *APOC3* gene polymorphisms influence TG levels by affecting *APOC3* function, and the elevation of TG may, in turn, promote the development of MASLD. Two underlying mechanisms may explain how polymorphisms modulate APOC3 activity. Firstly, the polymorphic allele enhances the transcriptional activity of *APOC3*, resulting in higher plasma *APOC3* levels [34]. Another hypothesis posits that *APOC3* mutation may directly regulate the expression of *APOC3* by affecting microRNAs’ binding to the 3' untranslated region of APOC3. However, further research is needed to confirm these findings [35, 36].

This study presents several limitations that must be acknowledged. First, although the sample size of 440 participants provides a foundation for analysis, it may be too small to detect subtle genetic effects or interactions. Second, the cross‐sectional nature of the study limits our ability to infer causality, and the lack of detailed analysis of lifestyle factors such as diet and physical activity may also have potential impacts on the results. Third, this study is conducted among the Chinese population, which may limit the applicability of the findings to other ethnic groups. Future studies should expand the sample size to include multiethnic populations, adopt longitudinal designs to enhance causal inference and experimentally validate genetic effects to strengthen the causal understanding of disease mechanisms.

## 5 Conclusions

In conclusion, *APOC3* rs5128, rs2854116 and rs2854117 polymorphisms are associated with MASLD risk, with TG serving as a potential mediating factor. *ANGPTL*8 rs2278426 polymorphism is not associated with MASLD.

## Author Contributions

**Jia Pan:** conceptualization (lead), data curation (lead), formal analysis (lead), funding acquisition (equal), investigation (equal), methodology (lead), project administration (lead), resources (lead), writing – original draft (lead), writing – review and editing (lead). **Xue Wang:** conceptualization (supporting), data curation (supporting), formal analysis (supporting), investigation (supporting), writing – original draft (supporting). **Youjin Zhang:** conceptualization (supporting), data curation (supporting), formal analysis (supporting), investigation (supporting), methodology (supporting). **Ting Wang:** conceptualization (supporting), formal analysis (supporting), investigation (supporting), methodology (supporting). **Saiqun Lv:** conceptualization (supporting), data curation (supporting), investigation (supporting), methodology (supporting). **Xiaoli Zhang:** conceptualization (supporting), data curation (supporting), formal analysis (supporting), funding acquisition (supporting). **Yuanyuan Zhou:** conceptualization (supporting), data curation (supporting), methodology (supporting). **Tao Peng:** conceptualization (equal), data curation (supporting), investigation (equal), methodology (supporting), writing – review and editing (lead). **Yongyan Song:** conceptualization (lead), data curation (supporting), methodology (lead), writing – review and editing (lead).

## Ethics Statement

The study was approved by the ethics committee of the Clinical Medical College & Affiliated Hospital of Chengdu University (PJ2024‐097‐02) and was carried out in accordance with the Declaration of Helsinki. Informed consent was obtained from all participants.

## Conflicts of Interest

The authors declare no conflicts of interest.

## Supporting information


**Supplementary 1**.

## Data Availability

The data that support the findings of this study are available from the corresponding author upon reasonable request.
