# Supplementary material for: Associations Between APOC3 and ANGPTL8 Gene Polymorphisms With MASLD Risk and the Mediation Effect of Triglyceride on MASLD in the Chinese Population
Source: J Cell Mol Med. 2025 Apr 7;29(7):e70542. doi: 10.1111/jcmm.70542 (PMC11974264; doi:10.1111/jcmm.70542)
Supplement: Supplementary file 1 — Supplementary 1. [file JCMM-29-e70542-s001.docx]

Supplemental Materials: PCR and Polymerase Chain Reaction-Restriction Fragment Length Polymorphism (PCR-RFLP) Protocols

# **Section 1 PCR protocol**

## 1 Prepare the Reaction Mix

In a PCR tube, combine the following components:

- 2 µL DNA template
- 1 µL forward primer
- 1 µL reverse primer
- 12.5 µL 2xTaq PCR mix（Tiangen, KT201）
- Nuclease-free water to a final volume of 25 µL.

## 2 Set Up the PCR Program (Thermo Fisher Scientific Applied Biosystems SimpliAmp Thermal Cycler)

Program the thermal cycler with the following steps for *APOC*3 SNPs (rs5128, rs2854116 and rs2854117) and *ANGPTL*8 rs2278426**:**

- Initial Denaturation: 95°C for 7 minutes.
- Cycling (35 cycles):

Denaturation: 95°C for 30 seconds.

Annealing: 63°C for 30 seconds.

Extension: 72°C for 40 seconds.

- Final Extension: 72°C for 8 minutes.
- Hold: 4°C indefinitely.

## 3 Run the PCR

Place the PCR tubes in the thermal cycler and start the program.

## 4 Analyze the PCR product

- Run the PCR product on a 3% agarose gel at 120V for 30 minutes in 1x TAE buffer.
- Visualize under UV light to confirm the expected band size: *APOC*3 rs5128: 428bp; *APOC*3 rs2854116 and rs2854117: 234bp; *ANGPTL*8 rs2278426: 387bp.

# **Section 2 polymerase chain reaction-restriction fragment length polymorphism (PCR-RFLP) protocol for *APOC*3 rs5128**

## 1 Digest the PCR Product

In a PCR tube, combine the following components (Restriction enzyme is added last):

- 1 µg PCR product (from Section 1)
- 2 µL SacI restriction enzyme (Thermo Fisher Scientific, ER1135)
- 2 µL 10x Buffer SacI (Thermo Fisher Scientific, ER1135)
- Nuclease-free water to a final volume of 30 µL

## 2 Incubate the reaction mix

Place the reaction mix in a thermal water bath at 37°C and incubate overnight.

## 3 Analyze the digested PCR product

- Run the digested PCR product on a 3% agarose gel stained with GelRed at 120V for 30 minutes in 1x TAE buffer.
- Visualize under UV light to confirm the expected band size: *APOC*3 rs5128-CC genotype: 428bp; GG genotype: 308bp+120bp; GC genotype: 428bp+308bp+120bp.

# **Section 3 polymerase chain reaction-restriction fragment length polymorphism (PCR-RFLP) protocol for** ***ANGPTL*8 rs2278426**

## 1 Digest the PCR Product

In a PCR tube, combine the following components (Restriction enzyme is added last):

- 1 µg PCR product (from Section 1)
- 1 µL BtsCI restriction enzyme (New England Biolabs, R0647S)
- 5 µL 10x Buffer BtsCI (New England Biolabs, R0647S)
- Nuclease-free water to a final volume of 50 µL

## 2 Incubate the reaction mix

Place the reaction mix in a thermal water bath at 50°C and incubate overnight.

## 3 Analyze the digested PCR product

- Run the digested PCR product on a 3% agarose gel with GelRed at 120V for 30 minutes in 1x TAE buffer.
- Visualize under UV light to confirm the expected band size: *ANGPTL*8 rs2278426-CC genotype: 387bp; CT genotype: 387bp+331bp+56 bp; TT genotype: 331 bp+56 bp. The 56 bp band may not be visible on a 3% agarose gel due to its small size.
